# Supplementary material for: Interactions between the microbiome and mating influence the female’s transcriptional profile in Drosophila melanogaster
Source: Sci Rep. 2020 Oct 23;10:18168. doi: 10.1038/s41598-020-75156-9 (PMC7584617; doi:10.1038/s41598-020-75156-9)
Supplement: Supplementary file 1 — Supplementary Figures. [file 41598_2020_75156_MOESM1_ESM.pdf]

Interactions between the microbiome and mating influence the female's transcriptional profile in *Drosophila melanogaster*

Sofie Y. N. Delbare, Yasir H. Ahmed-Braimah, Mariana F. Wolfner, Andrew G. Clark

Supporting Information

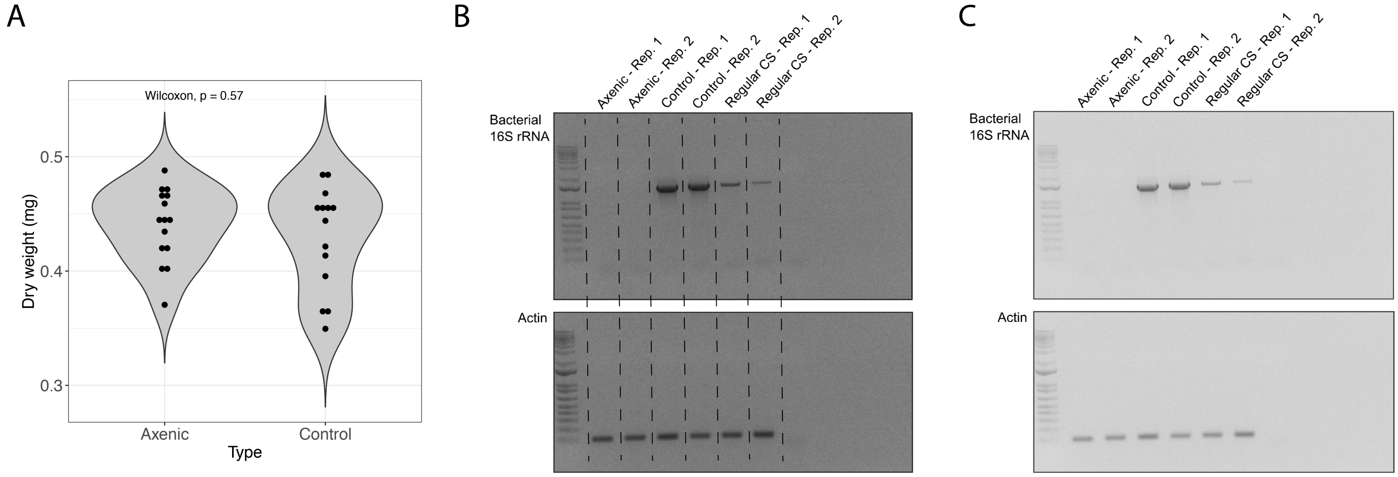

Figure S1. A: Comparison of dry weight between axenic and control females (axenic $n = 15$; control $n = 14$).B: Modified gel image of PCR assay to verify absence of bacteria in axenic flies and presence of bacteria in control. A mix of 9-12 pooled adult females and males was used for each sample on this gel. Axenic = dechorionated Canton-S flies with sterile 1X PBS added to sterile food. Control = dechorionated Canton-S flies with fly homogenate added to sterile fly food. Regular = untreated Canton-S flies. Rep. = biological replicate. Samples with primers for bacterial 16S rRNA and Actin (positive control) were run on the same gel, but imaged separately for clarity. C: Original gel image in "B" prior to brightness/contrast modification.

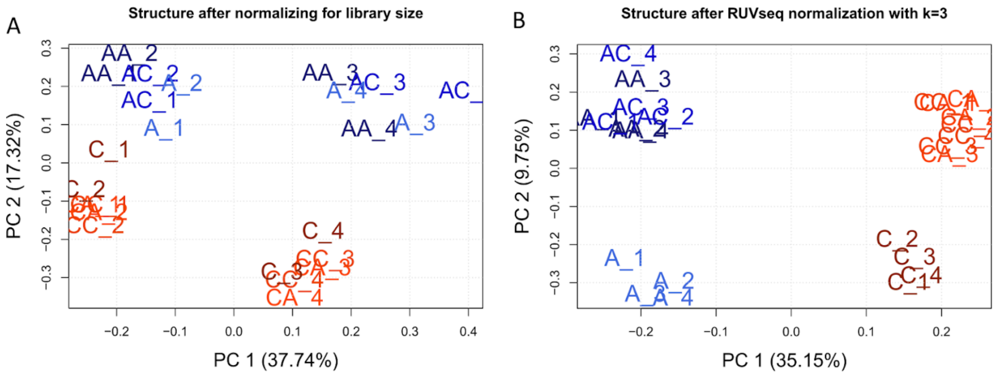

Figure S2. Principal Component Analysis of RNA-seq samples. A: Before adjustment for batch effect using RUVseq. B: After adjusting for batch effect using RUVseq. AA = axenic female mated to axenic male; AC = axenic female mated to control male; CA = control female mated to axenic male; CC = control female mated to control male, C = control virgin female, A = axenic virgin female. Numbers indicate the batch (biological replicate).

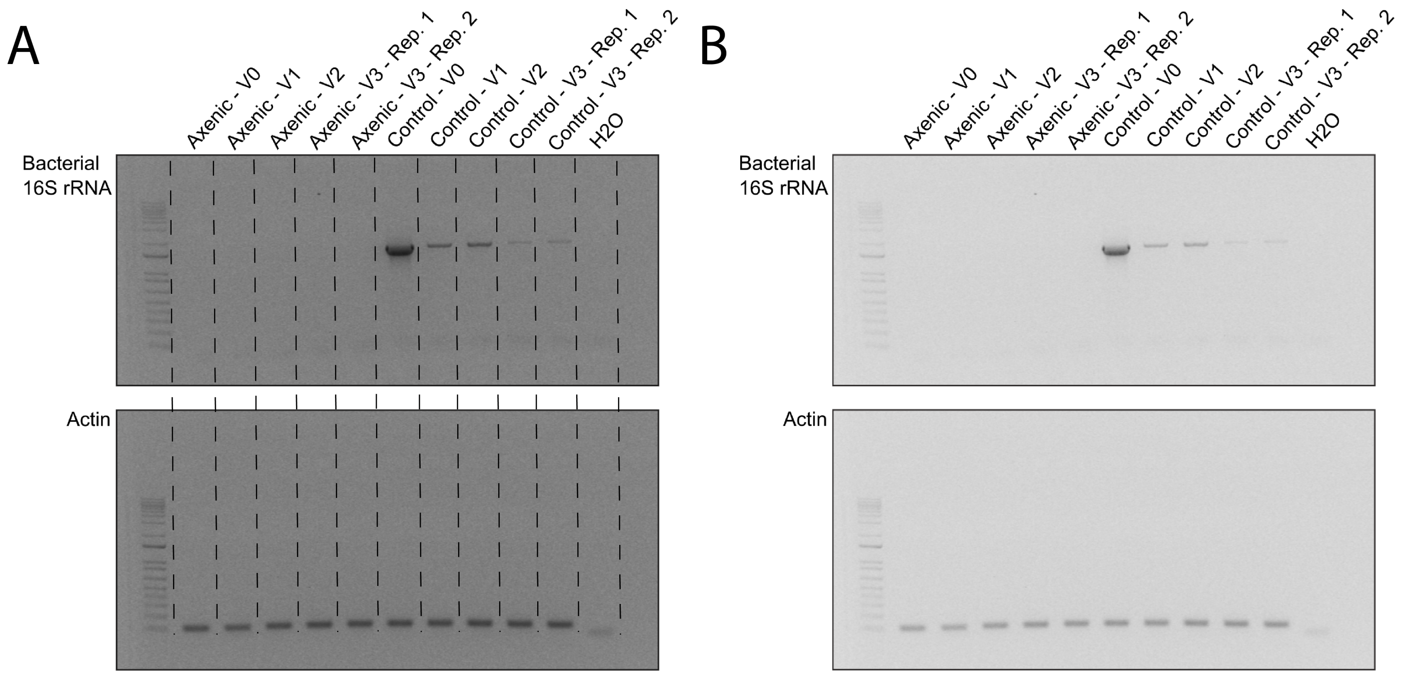

Figure S3. A: Modified gel image of PCR assay to verify the presence of bacteria in control flies and absence of bacteria in axenic flies used for the egg laying assay. 2-3 flies were pooled at each time point. V0-3 = vial 0 to 3. Rep. = biological replicate. Samples with primers for bacterial 16S rRNA and Actin (positive control) were run on the same gel, but imaged separately for clarity. B: Original gel image in "A" prior to brightness/contrast modification.

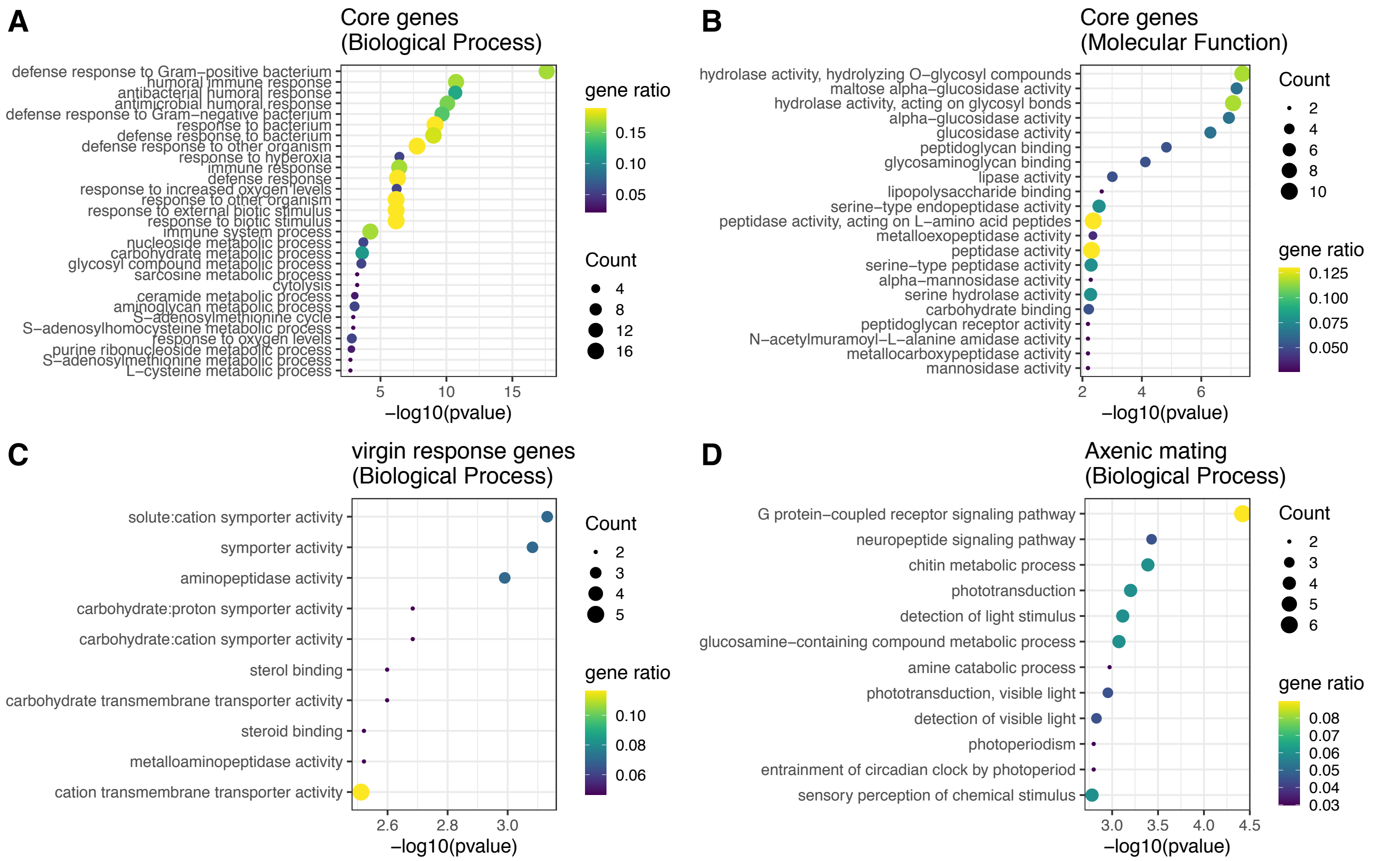

Figure S4. GO term enrichment analysis for differentially abundant transcripts. (A-B) Enriched GO terms for the 124 "core" genes whose RNA levels are influenced by the microbiome in both virgin and mated females. (C) Enriched Biological Process GO terms for 57 genes whose RNA levels are influenced by the microbiome in virgin females only. (D) Enriched Biological Process GO terms for 92 genes that are down-regulated in mated axenic females relative to mated control females. The gene ratio is (# of DE genes with GO term x) / (total # of genes with GO term x in the *Drosophila* genome).

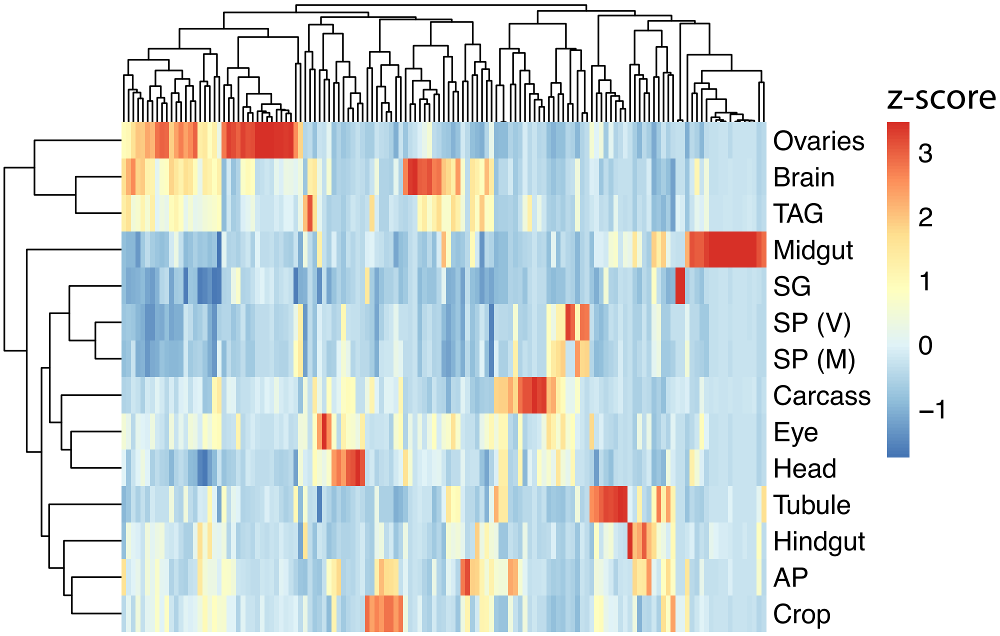

Figure S5. Enrichment values across 14 female tissues for genes whose transcript abundance is impacted in axenic females depending on the male's microbiome. Enrichment scores were calculated using TPM values from FlyAtlas2 [52]. The heatmap was generated with the R package pheatmap (v. 1.0.12). (AP = Anal plate; SP = spermathecae; M = mated; V = virgin; TAG = thoracico-abdominal ganglion)

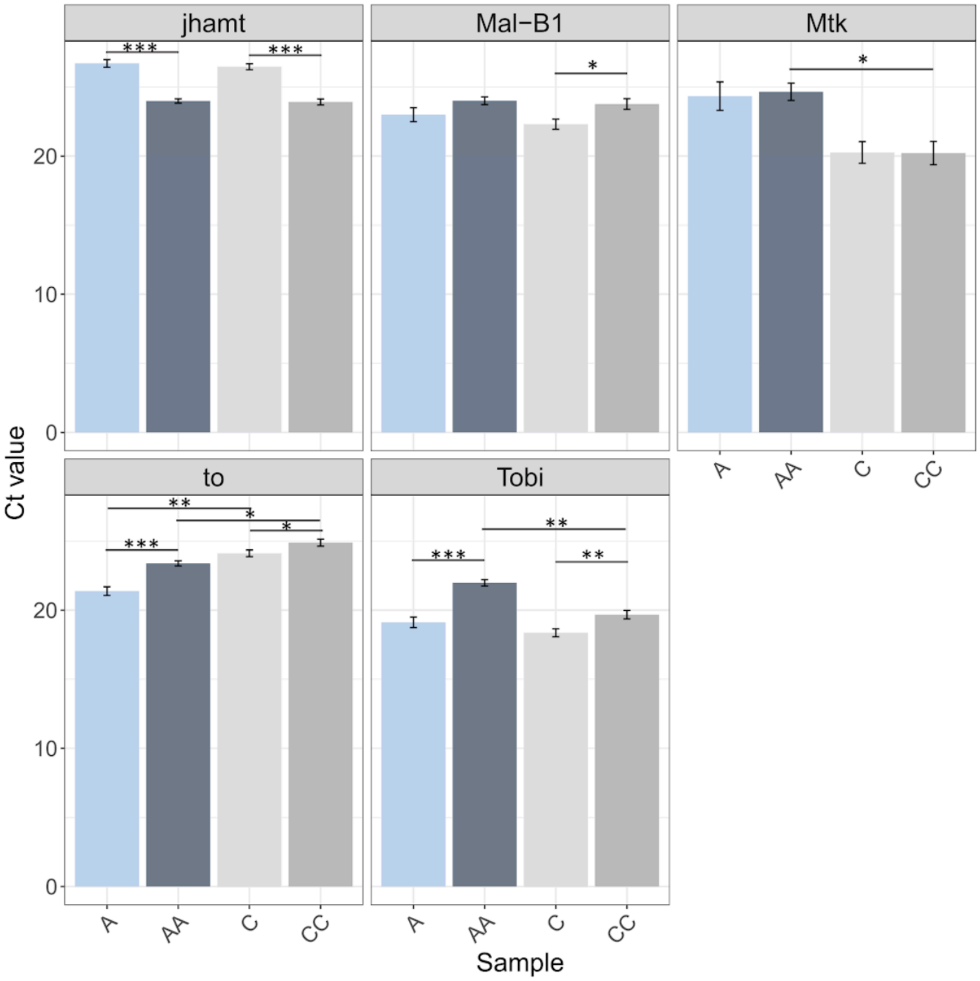

Figure S6. qRT-PCR validation of changes in transcript abundance observed in the RNA-seq analysis. Samples for qRT-PCR were collected independently from those used for RNA-seq. A = axenic virgin female; C = control virgin female; AA = axenic female mated to axenic male; CC = control female mated to control male. Bars show the estimated marginal means (EMM) of Ct values and standard errors of the EMM, based on linear models that were fitted for each gene independently. A higher Ct value indicates a lower expression of the gene. Each EMM was determined based on 3 biological replicates, which each contained 10 pooled females. All expression values were normalized to the housekeeping gene *Nervana*, except for *Mal-B1*, which was normalized against *Rp49*. Stars indicate significance of pairwise contrasts between the samples. * $p < 0.05$; ** $p < 0.01$; *** $p < 0.001$. If no star is shown, the contrast was not significant. After correcting for multiple testing using the Benjamini-Hochberg correction, no contrasts are significantly different for *Mtk*; for *jhamt*, both A-AA and C-CC have $p < 0.05$; for *to*, A-AA and A-C have $p < 0.05$; for *Tobi*, A-AA, AA-CC and C-CC have $p < 0.05$ and for *Mal-B1*, C-CC has $p < 0.05$.
